# A Current Overview of the *Papaya meleira virus*, an Unusual Plant Virus

**DOI:** 10.3390/v7041853

**Published:** 2015-04-08

**Authors:** Paolla M. V. Abreu, Tathiana F. S. Antunes, Anuar Magaña-Álvarez, Daisy Pérez-Brito, Raúl Tapia-Tussell, José A. Ventura, Antonio A. R. Fernandes, Patricia M. B. Fernandes

**Affiliations:** 1 Núcleo de Biotecnologia, Universidade Federal do Espírito Santo, Vitória 29040090, Espírito Santo, Brazil; E-Mails: paollaabreu1@gmail.com (P.M.V.A.); tathiana_antunes@hotmail.com (T.F.S.A.); anuar.magana@cicy.mx (A.M.-Á.); ventura@incaper.es.gov.br (J.A.V.); alberto.fernandes@ufes.br (A.A.R.F.); 2 Laboratorio GeMBio, Centro de Investigación Científica de Yucatán A.C., Mérida 97200, Yucatán, Mexico; E-Mails: daisypb@cicy.mx (D.P.-B.); rtapia@cicy.mx (R.T.-T.); 3 Instituto Capixaba de Pesquisa, Assistência Técnica e Extensão Rural, Vitória 29050790, Espírito Santo, Brazil

**Keywords:** papaya sticky disease, dsRNA genome virus, phytopathogenic virus, laticifers colonization

## Abstract

*Papaya meleira virus* (PMeV) is the causal agent of papaya sticky disease, which is characterized by a spontaneous exudation of fluid and aqueous latex from the papaya fruit and leaves. The latex oxidizes after atmospheric exposure, resulting in a sticky feature on the fruit from which the name of the disease originates. PMeV is an isometric virus particle with a double-stranded RNA (dsRNA) genome of approximately 12 Kb. Unusual for a plant virus, PMeV particles are localized on and linked to the polymers present in the latex. The ability of the PMeV to inhabit such a hostile environment demonstrates an intriguing interaction of the virus with the papaya. A hypersensitivity response is triggered against PMeV infection, and there is a reduction in the proteolytic activity of papaya latex during sticky disease. In papaya leaf tissues, stress responsive proteins, mostly calreticulin and proteasome-related proteins, are up regulated and proteins related to metabolism are down-regulated. Additionally, PMeV modifies the transcription of several miRNAs involved in the modulation of genes related to the ubiquitin-proteasome system. Until now, no PMeV resistant papaya genotype has been identified and roguing is the only viral control strategy available. However, a single inoculation of papaya plants with PMeV dsRNA delayed the progress of viral infection.

## 1. Introduction

Plant viruses cause many diseases of international importance and are responsible for huge losses of crop production and quality in all parts of the world. The production is impaired due to the susceptibility of the plants to the viruses. 

**Figure 1:**
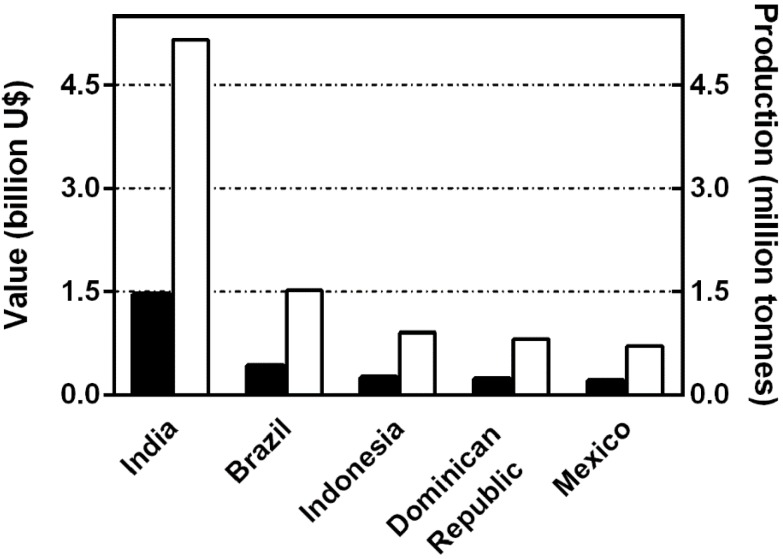
The graph represents the five highest papaya producing countries in 2012. Solid bars correspond to revenue of billions of US dollars and open bars, production in million tonnes.

The main producers of papaya are India, Brazil, Indonesia, Dominican Republic, Nigeria and Mexico, and combined they produce approximately 10 million tons per year [1] (Figure 1).

Brazil is one of the main papaya producers; in 2012, the country produced 1.52 million tons, which was approximately 12.2% of the world supply. The production area was 31.3 thousand hectares with an average yield of 48.5 t/ha, and the production value estimated at $ 786 million [1]. Mexico, on the other hand, is the main exporter to the USA. In 2013, it cultivated approximately 16,368 hectares, with a production of 764,514 tons [2]. The economic importance of this crop for both countries, besides the high levels of fruit exportation, is that this crop is the source of income for hundreds of smallholder farmers. 

Papaya viruses cause diseases of international importance with serious reductions in fruit production that may even totally destroy affected orchards. Until now, more than ten different viruses have been reported in papaya worldwide. Nevertheless, the most important viruses that affect papaya are the *Papaya ringspot virus* (PRSV), the *Papaya leaf distortion mosaic virus* (PLDMV), the *Papaya lethal yellowing virus* (PLYV), the *Papaya mosaic virus* (PapMV) and the *Papaya meleira virus* (PMeV), which have been known to cause serious damage to the crop production throughout the world. 

PRSV causes the most destructive viral disease of papaya crop, the papaya ringspot, and has been found in many tropical and subtropical areas where papaya is grown, including the USA, South America, Mexico and Japan [3]. PLDMV, was first reported in 1954 on the island of Okinawa, Japan. PLDMV emergence in PRSV-resistant papaya transgenic lines was considered as an emerging threat to papaya culture in China [4]. PYLV is restricted to Brazil and its increasing spread reaching high incidence rates [5]. PapMV was first reported in 1962 in Florida, USA. The disease has spread to other countries, reaching Bolivia, Peru, Venezuela and Mexico. In Mexico, PRSV and PapMV occurred in single or mixed infections and a synergistic interaction between the two independent viruses in the same host can occur and lead to increased symptoms and virus accumulation [6].

In Brazil, the percentage of plants eradicated with papaya ringspot is about 2% per year in well-managed orchards. In traditional orchards, the losses may reach 80% [7]. In Mexico, PRSV causes severe damage in the main papaya production states with crop losses of up to 85% [8]. The PMeV infects at least 20% of the plants during the economic cycle of crop. In some orchards, where rouging of sticky diseased plants was not carried out, an incidence of the disease of up to 100% and cause total yield losses [9]. 

Little information exists about PYLV and its economic importance for fruit production, but in some orchards the disease may have an incidence of up to 40% of the plants. The disease caused by PLDMV has low economic importance to the papaya production in Brazil [7], and in Mexico this disease has not be found.

Little has been published in the patent databases concerning methods for the detection of the *Papaya meleira virus*, as well as on strategies for eradicating the papaya sticky disease. A search in the Derwent Innovations Index yielded approximately 160 patents related to papaya viruses, but most of these patents involved the *Papaya ringspot virus* or the *Papaya mosaic virus*. In our view, research groups all over the world face a significant opportunity to reduce production losses in papaya cultivation by doing basic research on papaya infestation by PMeV.

## 2. Papaya Meleira Virus

PMeV, the causal agent of Papaya sticky disease or “meleira”, was reported in the 1980s in Brazil [10], and in 2008, it also appeared in Mexico [11]. Although PMeV is considered one of the major viruses infecting papaya in Brazil and in Mexico, knowledge of the sequence and genomic organization of this virus is poor. PMeV has not been sequenced nor classified by the International Committee on the Taxonomy of Viruses (ICTV). A comparative analysis of a ~560 bp fragment amplified from the PMeV replicase gene from the major Brazilian papaya-producing states isolates indicated that PMeV possesses a similarity with the mycoviruses of the family Totiviridae [12,13]. However, a conclusive taxonomic classification will only be possible when the full genome is sequenced. PMeV is an isometric virus particle that has a double-stranded RNA (dsRNA) genome of 12 Kb [14,15]. Papaya fruits with sticky disease symptoms are commercially unacceptable as the disease compromises their texture and flavor, rendering them unfit for consumption, which prevents their exportation to the international market [9]. 

## 3. Etiology

**Figure 2:**
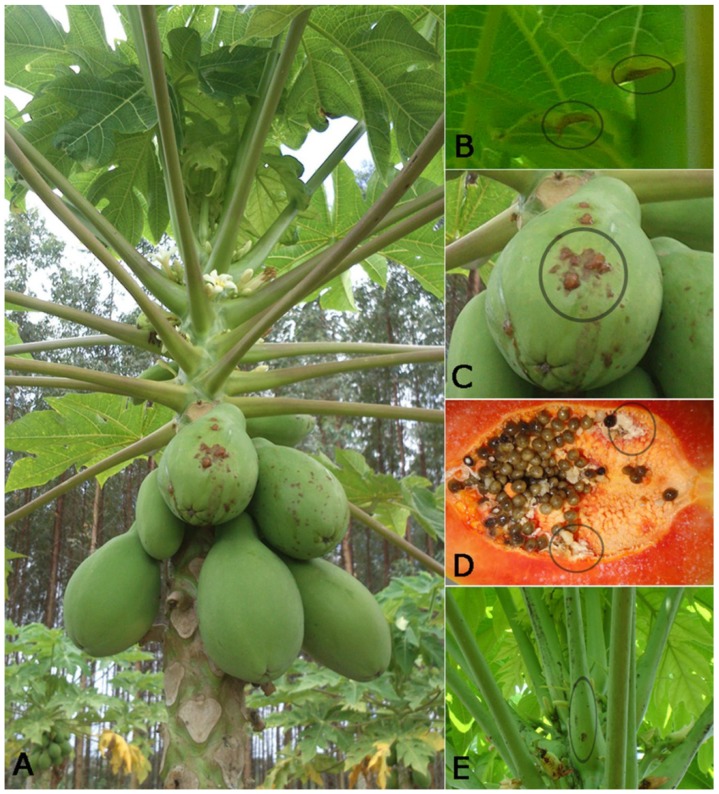
Symptoms of PMeV infection in *Carica papaya*. Papaya plant showing the symptoms of sticky disease (**A**). Plants spontaneously leak the latex, which oxidizes after atmospheric exposure resulting in small necrotic lesions on the edges of young leaves (**B**) and fruits with sticky aspect (**C**). Additionally, diseased plants in Mexico show latex inside of the fruit cavity (**D**) and necrotic spots in the petioles (**E**). The black circles highlight the main symptoms.

Papaya sticky diseased affected plants are characterized by spontaneous exudation of fluid and aqueous latex from the fruit and leaves. The latex oxidizes after atmospheric exposure, resulting in small necrotic lesions on the edges of young leaves (Figure 2A) and a sticky appearance of the fruit from which the name of the disease originates (Figure 2B) [9]. In advanced stages of the disease, irregular light-green areas have been observed on the surface of the infected fruits in Brazil; however, this symptom has not been commonly observed in Mexico. In addition to these symptoms, diseased plants in Mexico show small internal blotches in the pulp of the diseased fruit, necrotic spots in the petiole (Figure 2C) and, in the most severe cases, the presence of latex inside the fruit cavity covering the seeds was also observed (Figure 2D) [11].

Initially, symptoms of the disease were attributed to a disturbance in the calcium and boron absorption that resulted from water stress or an imbalance of these elements in the soil [16]. In addition to the abiotic factors, the involvement of microorganisms was suggested after the isolation of a bacteria of the genus Bartonella from the diseased plants [17]. 

Monitoring the sticky disease dispersal in commercial orchards indicated a pathogen involvement [10]. The biotic etiology of this disease was verified in preliminary studies where healthy papaya plants that were injected with a latex obtained from the diseased plants developed the anticipated typical symptoms as early as 45 days after inoculation, suggesting that the causative agent was present in the plant latex [10]. Finally, the viral etiology of the disease was confirmed after the purification of viral particles that were present in the latex and that was followed by the inoculation of these particles in the healthy papaya seedlings, which developed typical sticky disease symptoms [15]. Transmission electron microscope studies of the diseased plant leaves and fruit latex samples indicated the presence of a large number of isometric particles of approximately 50 nm in diameter. Ultrafine tissue sections revealed that these particles were restricted to the lactiferous vesicles [14]. 

The viral nucleic acid with approximately 12,000 base pairs was extracted from purified viral particles and visualized in an agarose gel [14,15]. The genetic material composition was proven to be RNA by its resistance to DNase digestion and susceptibility to RNase A digestion. In order to confirm whether the viral genome was comprised of single-stranded RNA (ssRNA) or double-stranded RNA (dsRNA), it was digested with S1 nuclease that degrades ssRNA. As a result, the viral nucleic acid was resistant to S1 nuclease, and it was concluded that *Papaya meleira virus* is a dsRNA genome virus [15]. 

Viruses with a dsRNA genome are unusual among plant viruses, which have, in most cases, a genome consisting of single stranded RNA (ssRNA). dsRNA viruses represent a small group among plant viruses, and they are grouped in the Endornaviridae, Partitiviridae and Reoviridae families [18].

**Figure 3:**
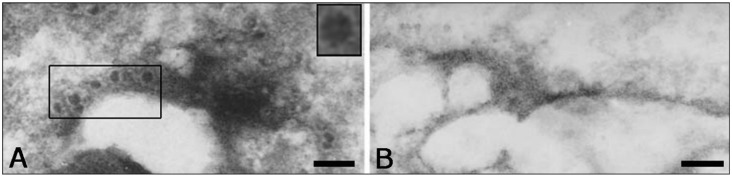
Papaya latex analysis by transmission electron microscopy (TEM). (**A**) Diseased and (**B**) healthy latex samples were studied by TEM, which revealed the PMeV particles (highlight) on the polymers. Inset show the amplified virus particle. The scale bar is 200 nm on both images.

Moreover, unusually for a plant virus, PMeV appears to reside primarily in the laticifers, which is an inhospitable environment, where it the modifies potassium levels and the osmotic balance that leads to cellular rupture [19]. The results obtained from the electron microscopy and molecular analyses indicated that the viral particles were localized on [14] and linked to the polymers present in the latex, perhaps acting as a protective mechanism or in assisting the viral transport (Figure 3) [19].

## 4. Epidemiology

Sticky disease spreads rapidly, and currently it occurs mostly in Northeast Brazil [12]. Outside Brazil, Mexico is the only other country where the disease was reported [11]; at this time, the virus continues its dissemination and it has been detected in all producer states and its incidence in papaya orchards has been increasing. 

**Figure 4:**
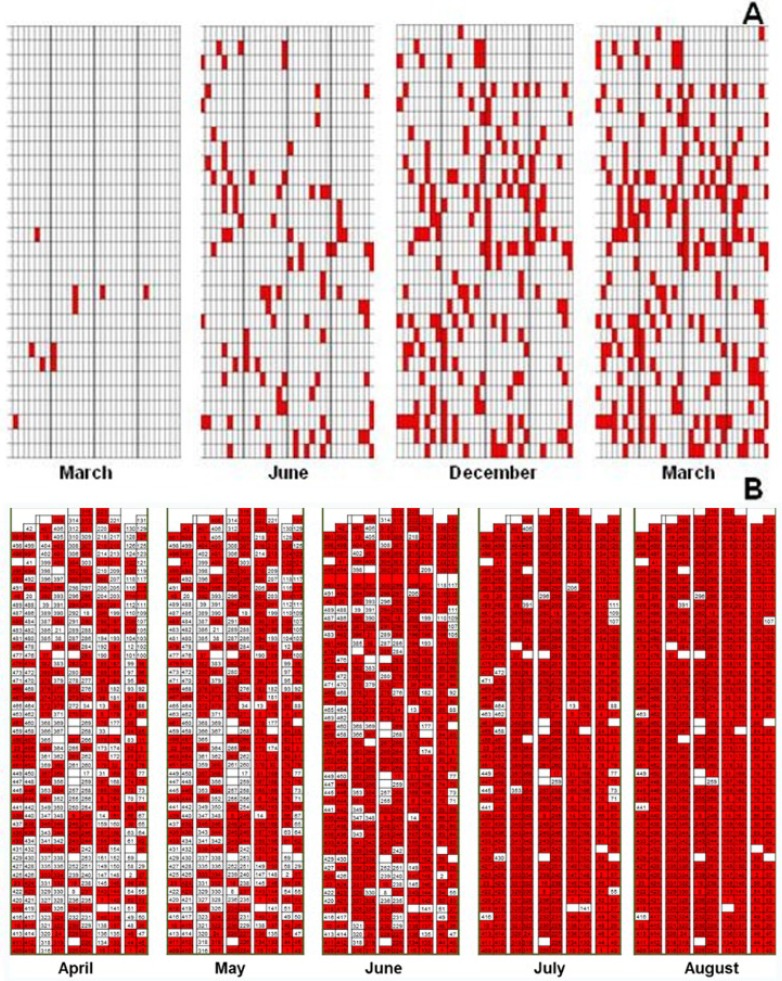
Spatial pattern maps of PMeV on papaya orchards in Brazil (**A**) and in Mexico (**B**). Red squares represent diseased plants and white represent healthy ones.

Agricultural practices are responsible for the spread of the disease within the orchard because the dispersion of the sticky disease often occurs along the array of the crop row [9]. Therefore, laboratory experiments were conducted in order to test five different mechanical inoculation methods that were designed to simulate injuries caused by work tools and the movement of vehicles in the orchard. Among the tested methods, cutting the leaf, cutting the leaf stalk, scraping the leaf surface, scraping the stems and the injection into the stem apex only the injection of infected latex into the stem apex resulted in plant infection [20]. In Mexico, the PMeV spatial distribution was studied in experimental plots over a year, and molecular diagnosis was performed at intervals of 21 days on all of the evaluated plants. The high percentage of infected plants (approximately 78%) per line during and after the harvest pointed to mechanical transmission [21], and this result corroborated the findings of Ventura *et al.* in 2003 [9] (Figure 4). For that reason, we may conclude that among agricultural practices, fruit thinning is responsible for the spreading of sticky disease.

As for the progress of the disease with time, the epidemiological behavior varied according to different factors, such as environmental factors, possible vectors and cultural practices. A positive correlation has been observed between the temperature and precipitation in the course of the disease [21,22]. In the field, the disease occurred mainly in two epidemic patterns according to the Logistic and Gompertz models (*R*^2^ > 0.98), which influenced the culture lifespan permanence [22]. In general, the first symptoms of the sticky disease appeared in plants that were 6 to 9 months old, depending upon the papaya variety. The initial onset of the incidence of sticky disease varied according to the source of inoculum, from seeds or alternative hosts and vectors, which played an important role in the quality of the papaya seedlings’ health [9,23].

Identifying the mode of transmission of the papaya sticky disease has been an issue. Abreu *et al.* [24] investigated the PMeV transmission by *C. papaya* cultivar Golden seeds. They also developed a method for the diagnosis of the viral infection in seedlings. No PMeV was detected by conventional RT-PCR on the numbers of seedlings that were derived from the seeds of healthy (*n* = 187) and infected (*p* = 172) plants. However, another study conducted with seeds from *C. papaya* cultivar Maradol demonstrated that PMeV is seed-borne and that it can be transmitted to the next generation via contaminated seeds. Furthermore, this finding may explain how this virus appeared in Mexico [23]. The frequency of virus transmission by seeds can vary greatly depending on the virus-host interaction, and may be very small in some cases [25,26]. Therefore, it may vary according to the papaya cultivar, being very low for *C. papaya* cultivar Golden that requires a larger number of seedlings in order to be analyzed, and it is very high for *C. papaya* cultivar Maradol accounted for the high incidence of PMeV in Mexico. Moreover, for the seed transmission of plant viruses, the seed might be infected in the generative cells and the virus is maintained in the germ cells or, less often, in the seed coat [27]. Papaya seeds are extremely wrinkled, and each seed is enclosed in a gelatinous membrane, and, with fruits that are infected with the sticky disease, soaked with infected latex. For this reason, it is still necessary to confirm whether PMeV is in the seed embryo or in the outside shell.

It is worth mentioning that it is extremely important to conduct surveys in other papaya producing countries of the region and with the major producers worldwide in order to verify the virus presence in these areas, especially given the increase in the movement of seeds among the producing countries.

The possible involvement of insects as sticky disease vectors has been considered based upon early studies on the field spread pattern of this disease [10,28], especially with evidence of the existence of an aerial vector associated with the disease. It was shown that the presence of dsRNA and sticky disease symptoms occurred six and eight months, respectively, after healthy papaya plants were exposed to PMeV-infected whitefly (*Bemisia tabaci* type B) [29], although the virus was not detected in this possible vector, which is not a papaya pest. On the other hand, adults of another whitefly species, *Trialeurodes variabilis*, a pest to Brazilian papaya and other fruit trees, [30] possess PMeV dsRNA. Nevertheless, despite large populations of the whitefly, no virus infection was observed in those plants, including plants exposed to insects after they fed on PMeV inoculated plants, suggesting that the insect acquires the virus but does not transmit it to papaya plants [31]. A greenhouse study confirmed that *T. variabilis* was unable to transmit PMeV from diseased to healthy papayas, even though the presence of the virus was ascertained in adults and nymphs [20]. Currently, research teams in Brazil and Mexico are undertaking research to identify the virus vector and elucidate the mechanism of transmission. 

Sticky disease also interferes with the natural resistance of papaya fruits to fruit flies (Diptera: Tephretidae). Green unripe fruits from PMeV infected plants are susceptible to the Mediterranean fruit fly (Medfly), *Ceratitis capitata* (Wied.), a pest of quarantine importance that usually only infests papaya fruits in the advanced stages of maturity. There is a direct relationship between the time of plant infection with the virus and the infestation of the papaya fruits by the fruit fly *C. capitata* [32].

Another important aspect in plant disease epidemiology is the alternative hosts. In this sense, the presence of dsRNA with a molecular weight similar to that associated with sticky disease was detected in samples of the Gramineae species *Brachiaria decunbens*, which is present in the papaya orchards that are affected by the sticky disease in the south of Bahia [15]. 

## 5. Detection Methods

Aiming for the earlier identification of infected plants, two different molecular approaches have been developed. The first strategy is based upon the extraction of PMeV genomic dsRNA from the plant latex using organic solvents followed by gel electrophoresis to visualization the resultant ~12 kb viral dsRNA band [33]. This method has been used both to analyze symptomatic plants and to confirm the field diagnosis. This has the advantage of relatively low cost and rapid turnover, but requires large quantities of latex and a high titre of the virus, making it unsuitable for the detection at the early stages of infection or when only a little amount of the plant material is available.

The second method applies conventional reverse transcription PCR (RT-PCR) to latex diluted in ammonium citrate. Viral dsRNA was used as a template in RT-RAPD (Reverse Transcription-Random Amplified Polymorphic DNA) to randomly produce DNA sequences from the PMeV genome. RAPD does not require prior DNA sequence information because it uses single, short random primers so it can be applied to generate random DNA sequence data from an uncharacterized virus RNA genome. RT-RAPD fragments were cloned and sequenced. The nucleotide sequences of the viral dsRNA were used to designed specific primers for PMeV. Through RT-PCR, one of these primers (C05-3) amplified a 669-nucleotide fragment that, after sequencing, showed a high similarity to other viral RNA dependent RNA polymerases (RDRP) [13]. This method requires less latex and is more sensitive, but the latex storage and latex proteins in the PCR reaction are problematic. 

The efficiency of either molecular diagnosis strategy, the direct PMeV dsRNA extraction or the amplification by RT-PCR, depends upon previous latex processing. Usually papaya producers send fruits, leaves and stems inside plastic bags to diagnostic laboratories. Sometimes the conditions during plant transport makes diagnosis from the latex impracticable as it must be correctly stored and processed to avoid dsRNA destruction and consequent diagnosis failure. Rodrigues and colleagues [33] evaluated different procedures for latex storage prior to PMeV molecular diagnosis. The results showed that PMeV dsRNA was protected for 25 days when the latex was diluted in a citrate buffer pH 5.0 (1:1 v/v) and maintained at −20 °C. At the same temperature, some protection was observed for pure latex or for latex diluted in ultra-pure water. However, the dsRNA was almost completely degraded after 25 days when it was maintained at 25 °C, which indicated the need for freezing [33]. 

New diagnostic methods have been proposed using conventional and quantitative RT-PCR (qRT-PCR), allowing for the molecular diagnosis in papaya tissues [24]. Two primers pairs, each pair (forward and reverse) was selected specifically for conventional RT-PCR or qRT-PCR reactions, were designed by targeting the PMeV sequence homologous to RDRP [24]. These methods have allowed for the identification of asymptomatic plants infected by PMeV in the field. In Mexico, primers were also developed for PMeV diagnostics using RT-PCR that was capable of detecting both isolates from Mexico and from Brazil and it was sensitive enough to detect the virus in any type of plant tissue and in asymptomatic plants [34]. 

## 6. Disease Management

Until now, a papaya genotype that is resistant to PMeV has not been identified despite all of the efforts coming from Brazil and Mexico. Symptoms are triggered only after flowering and, therefore, an infected plant without fruit or symptoms can remain unnoticed for months in the field acting as an inoculum source until it is finally detected and eliminated [7,9]. Thus, roguing of infected plants is the only available strategy to control this viral disease [35,36]. In Brazil, this practice is governed by Normative Instruction number 17, 27 May 2010, of the Brazilian Ministry of Agriculture, Livestock and Supply (MAPA). Weekly inspections are performed in the entire culture crop and plants with symptoms similar to those caused by PMeV are removed. In both countries, with the goals of reducing sticky disease dissemination as well as the prevention or the delaying of its introduction into areas where the disease has not been noted, the following measures have also been recommended: (i) to conduct weekly inspections in orchards and eliminate diseased plants (roguing) as soon as the first symptoms of the sticky disease are detected [7,9]; (ii) to install nurseries and new orchards as far as possible from other orchards, especially those with a history of sticky disease; (iii) not to collect seeds from sticky diseased plants and orchards with PMeV occurrence [23]; (iv) to try to reduce injuries to plants during cultural treatments and disinfect all material used in the process of thinning and harvesting fruits; (v) to manage low-growing vegetation and keep rows clean and the area between rows trimmed to diminish the variety of weed species; (vi) to eliminate all orchards (diseased and healthy) at the end of the economic cycle of production to eliminate sources of inoculum; (vii) to destroy abandoned orchards, mainly those with plants infected by PMeV as one diseased papaya tree can infect the entire orchard and (viii) to implement crop rotation in papaya producing areas.

## 7. Papaya and PMeV Interaction

Papaya latex is mostly comprised of cysteine proteinases in addition to the carbohydrates, lipids, salts, phenols and glutathione [37]. These proteinases are synthesized as inactive precursors that are converted into mature enzymes after tissue wounding and latex exudation [37]. The PMeV’s ability to inhabit such a hostile environment shows an intriguing interaction with papaya.

PMeV particles are found only in papaya laticifers and not in other cell types [14]; therefore, the sticky disease symptoms, spontaneous latex exudation from fruits and leaves, might be a direct effect of the virus [19]. The characterization of the effects of the PMeV on papaya latex demonstrated that the accumulation of calcium oxalate crystals in the infected papaya latex positively correlated with the increased production of hydrogen peroxide (H_2_O_2_) in the laticifers of the plant, indicating a hypersensitive response (HR) of papaya laticifers against PMeV infection [19]. 

Latex fluidity and exudation in PMeV infected papaya suggest that the virus uses the host laticifers to move itself through the plant [19]. Electron microscopy and molecular data show the virus in close association with the latex particles. This association may allow the virus to flow more efficiently because the PMeV might use this mechanism to move quickly from the inoculation site to non-infected cells. This study also demonstrated alterations on lipids, phenols, alkaloids and sugar concentration occurring in papaya latex during PMeV infection [19]. 

Papaya latex is known to be mainly composed of proteins [38,39]; for this reason, the effects of PMeV on the laticifers’ regulatory network were addressed through the proteomic analysis of papaya latex. 160 unique papaya latex proteins were identified, where the majority of the proteins were associated with different types of stresses (e.g., cadmium ion, programmed cell death, drought, salinity, and microorganisms) [40]. Thus, papaya laticifers are involved in other plant stress responses in addition to the known herbivorous toxicity [37,41,42] and tissue protection after mechanical wounding [37,43]. Quantitative analysis revealed 10 down regulated proteins in the latex of diseased plants, including 9 cysteine proteases (chymopapain), showing that the PMeV influences the level of the proteases known to be activated during latex exudation [40]. 

Moreover, a reduction in the proteolytic activity of papaya latex during sticky disease has also been demonstrated, specifically associated with cysteine proteases, showing that these proteins are important in the response of the papaya to stress and that PMeV is able to overcome this process [40]. The reduced proteolytic activity seems to have an inhibitory effect on the latex coagulation by facilitating its flow through the laticifers, and, consequently, the virus spread within the plant [40]. Additionally, the accumulation of H_2_O_2_ in the laticifers of the infected papaya [19] played a negative regulatory role on the cysteine-protease activity during the sticky disease [37,44], most likely by oxidizing and inactivating the active site of the enzyme. Taken together, these results suggest the negative modulation of cysteine proteases of papaya latex by PMeV infection in an attempt to delay the process of the laticifers’ programmed cell death (PCD) [45] and the minimization of the virus particle degradation.

A major defense mechanism in plants is the “hypersensitive response” (HR) whereby cells infected with pathogens, and often surrounding cells as well, are instructed to self-destruct by the host plant [46]. This is thought to deny nutrients to the invading pathogen. It is proposed that in plants, the process is initiated by a reaction between nitric oxide (NO) and hydrogen peroxide (H_2_O_2_) [47]. The increased H_2_O_2_ production in the papaya laticifers during PMeV infection has already been demonstrated [19]. To assess the involvement of nitric oxide (NO) in plant defense signaling, papaya seedlings were challenged with a NO donor, sodium nitroprusside (SNP), and PMeV. SNP treatment led to the highest increase in peroxidase and superoxide dismutase activity, also the levels of phenolics and the carbohydrate concentration were also increased [48].

H_2_O_2_ is a well-known systemic response elicitor, and, as mentioned above, PMeV infected papaya laticifers have an increased production of this molecule. Additionally, necrotic lesions in the plant leaf tip is the first disease symptom [7,9]. Therefore, healthy and PMeV infected papaya leaves were analyzed using global protein expression analytical techniques (2-DE and DIGE) followed by protein identification by MS analysis (MALDI-TOF-MS/MS and LC-IonTrap-MS/MS) [49]. 

Stress responsive proteins, mostly calreticulin and proteasome-related proteins, are up regulated and proteins related to metabolism are down regulated in sticky diseased papaya leaf tissues [49], showing a major investment in plant defense [37]. In total, 42 up regulated and 33 down regulated protein spots were observed in the infected samples. Of those, 48 spots were identified, 26 being up regulated and 22 being down regulated [49]. 

The upregulation of proteasome-related proteins supports the assumption that PMeV proteins, or even host proteins, which are essential for the viral infection can be targeted for degradation [49]. Several studies have reported the involvement of the ubiquitin/26S proteasome system (UPS) in the signaling and regulation of the interactions between plants and pathogens [50,51,52], particularly with viral exploitation and interference with the UPS [53,54,55]. Therefore, these proteins are marker candidates useful for an additional analysis of papaya sticky disease to help better understand and control this disease [49]. 

Noncoding RNAs comprise the majority of transcribed RNA, and they perform a wide range of functions in cellular and developmental processes. As a result, noncoding RNAs are also implicated in the development and pathophysiology of many diseases, and they represent potential targets for therapeutic intervention. MicroRNAs (miRNAs) are one of a number of classes of endogenous, small (21–24 nucleotide), non-coding RNAs found in both animals and plants [56,57]. 

In the papaya, 24 [58] and 75 [59] conserved miRNAs were identified via the analyses of small RNA deep sequencing data and the genomic sequence. However, the conservation of mature miRNAs between plant species has enabled a computer-based approach to predict the secondary structures of the sequences surrounding miRNA. Actually, 4,251 plant mature miRNA sequences from the Plant miRNAs Database were used to search for hairpin structures in the *C. papaya* genome [60]. 462 known miRNA sequences were detected in 537 hairpins in the *C. papaya* genome; additionally, these miRNAs were classified into 72 miRNAs families [60].

Changes in miRNA expression are associated with viral infection in *Arabidopsis* [61], and viral protein-induced alterations in miRNA expression have been associated with symptom development in *Nicotiana tabacum* [62]. Therefore, in order to understand the unique plant-virus interaction between PMeV and *C. papaya*, and, consequently, the mechanisms involved in the onset of sticky disease symptoms, the miRNA response of the plant to infection was investigated [60]. The MicroRNAs that target the components of the 20S and 26S proteasomal degradation processes and components of other stress response pathways were studied. The expression of each was compared using real-time PCR in healthy and infected papaya leaf tissue [60]. The expression of these miRNAs changed, showing that PMeV can exploit and interfere with the UPS and with other stress response pathways [60]. PMeV modifies the level of several miRNAs involved in the modulation of genes related to the UPS system. The accumulation of these miRNAs in plants with sticky disease symptoms is lower, showing that PMeV takes over the UPS system for its own benefit, increasing its infectivity in papaya [60]. 

PMeV infection also influences the production of reactive oxygen species (ROS) [19,60]. This was particularly noticeable through the expression of target genes involved in the ROS pathway that played an important signaling role in the plants’ controlling processes, such as the response to biotic stimuli, which is lowered during PMeV infection in plants with sticky disease symptoms [60]. 

Therefore, evidence suggests that the PMeV uses the plant UPS response as a strategy to favor its replication and maintain host viability, and it modifies the accumulation of miRNAs that modulate important defense genes eliciting the onset of sticky disease symptoms [60].

## 8. Biotechnological Strategies to *Papaya meleira virus* Control

Efforts to control viral papaya diseases are long-standing. The currently used methods are vector control, the use of papaya plants that are resistant to certain viruses, and cross-protection, among others. Chemical or biological vector controls are used as well, and can be very effective considering vectors that need to feed for some time on a crop before the virus is transmitted, but are of much less value when the transmission occurs very rapidly and may already have taken place before the vector succumbs to the pesticide. As the sticky disease vector is still unknown, chemical or biological control cannot be used. The cross-protection strategy involves the use of a mild virus strain to control severe strains of the same virus. However, cross protection depends upon the availability of mild strains that can be used for effective protection against the target virus, and it requires extra agricultural practice and care that limits the benefits of this approach [3]. Until now, PMeV mild strains are unknown and, therefore, cross-protection is not a strategy that can be considered to control this virus.

Considering the lack of effectiveness of the methods mentioned above for PMeV control, the development of resistant papaya plants to viruses is the main goal, and virus-resistant transgenic plants are currently the most effective control of viral diseases.

Pathogen resistant plants can arise naturally or be developed by classic genetics or through the use of transgenic plants. The development of viral resistant varieties through conventional breeding methods has been complicated due to the sexual incompatibility of wild species and cultivated papaya [63]. 

A promising development in agricultural virus control is the concept that plants can initiate defense responses based upon RNA silencing when challenged by viral RNA. The use of transgenic plants that constituently express viral material has been successfully developed in different crops [64,65]. Genetic modified papaya resistant to certain viruses, for example to PRSV, is marketed in some countries. The concept of pathogen derived resistance (PDR) has been employed for PRSV management through coat protein (CP), RNA silencing, and replicase gene technology [3]. All of these gene technologies depend upon the viral genome sequence knowledge. Therefore, this methodology will only be considered after the complete PMeV genome has been sequenced.

**Figure 5:**
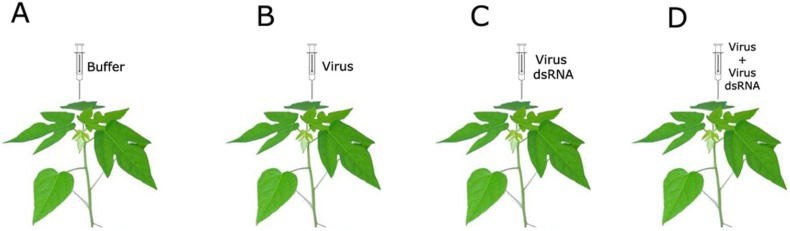
Induced resistance in papaya by viral dsRNA inoculation. Papaya seedlings were inoculated with (**A**) buffer (control); (**B**) PMeV; (**C**) PMeV dsRNA and (**D**) PMeV and PMeV dsRNA. A delay in the infection process occurred in the plants simultaneously inoculated by PMeV and PMeV dsRNA.

Alternatively, one way of inducing viral resistance in plants is to introduce artificially viral dsRNA molecules that are able to trigger the post-transcriptional gene silencing (PTGS) [66,67]. It has been shown that PMeV infected papaya plants inoculated with the dsRNA viral genome (Figure 5) have a delay in the infection process, suggesting that the plant defense has been elicited that has therefore inhibited viral replication [24]. Therefore, these results are a prospectus for sticky disease control.

Plant hormones may play a role in regulating signaling networks involved in plant defense. When a pathogen attack occurs, the plant produces these hormones, and the amounts and compositions vary depending upon the strategy of infection and the type of pathogen. At present, there has been significant progress in identifying the key components and the understanding of the role of phytohormones in plant responses to biotic attacks [68,69]. Studies on the papaya-PMeV interaction, such as those focused on the plant signaling pathways within the defense response, allow for a greater understanding of this mechanism and they open up the possibility for disease control by phytohormone-mediated papaya resistance [70,71].

## 9. Perspectives

Since the first report of the sticky disease in Brazil a few decades ago, many researchers have conducted several studies that have focused on different aspects of the disease; from this research, progress has been made in the molecular diagnosis of PMeV as well as in the knowledge of some of the mechanisms of transmission and spatiotemporal spreading of the virus. More recently, studies have focused on the proteomics of the most cultivated varieties of papaya in Mexico and Brazil, especially with a search for genes that are expressed in the presence of the virus because these genes can be molecular markers useful in searching for resistance to PMeV in papaya plants. Additionally, studies on the possible insect vectors for PMeV transmission have been conducted. More importantly, the virus genome is being sequenced as well.

To date, no variety of papaya has been found to be PMeV resistant. It is very important to focus on obtaining papaya plants that are resistant or tolerant to this virus through breeding programs that are based upon the information provided by the aforementioned studies or by the more effective genetic modification of papaya, which may be the best strategy for the efficient control of PMeV and for long-term protection.
